# Antibacterial Activity and Cell Responses of Vancomycin-Loaded Alginate Coating on ZSM-5 Scaffold for Bone Tissue Engineering Applications

**DOI:** 10.3390/ma15144786

**Published:** 2022-07-08

**Authors:** Z. Aslani, N. Nazemi, N. Rajabi, M. Kharaziha, H. R. Bakhsheshi-Rad, M. Kasiri-Asgarani, A. Najafinezhad, A. F. Ismail, S. Sharif, F. Berto

**Affiliations:** 1 Advanced Materials Research Center, Department of Materials Engineering, Najafabad Branch, Islamic Azad University, Najafabad, Iran; zahraaslanii777@gmail.com (Z.A.); nazemi_negar91@yahoo.com (N.N.); m.kasiri@pmt.iaun.ac.ir (M.K.-A.); aliakbar.najafinejad@gmail.com (A.N.); 2 Department of Materials Engineering, Isfahan University of Technology, Isfahan 84156-83111, Iran; rnegar29@gmail.com; 3 Advanced Membrane Technology Research Center (AMTEC), Universiti Teknologi Malaysia, Johor Bahru 81310, Johor, Malaysia; afauzi@utm.my; 4 Faculty of Engineering, Universiti Teknologi Malaysia, Johor Bahru 81310, Johor, Malaysia; safian@utm.my; 5 Department of Mechanical and Industrial Engineering, Norwegian University of Science and Technology, 7491 Trondheim, Norway

**Keywords:** zeolite (ZSM-5), three-dimensional scaffold, bone tissue engineering, antibacterial activity, bone mineralization

## Abstract

Despite the significant advancement in bone tissue engineering, it is still challenging to find a desired scaffold with suitable mechanical and biological properties, efficient bone formation in the defect area, and antibacterial resistivity. In this study, the zeolite (ZSM-5) scaffold was developed using the space holder method, and a novel vancomycin-loaded alginate coating was developed on it to promote their characteristics. Our results demonstrated the importance of alginate coating on the microstructure, mechanical, and cellular properties of the ZSM-5 scaffold. For instance, a three-fold increase in the compressive strength of coated scaffolds was observed compared to the uncoated ZSM-5. After the incorporation of vancomycin into the alginate coating, the scaffold revealed significant antibacterial activity against *Staphylococcus aureus* (*S. aureus).* The inhibition zone increased to 35 mm. Resets also demonstrated 74 ± 2.5% porosity, 4.3 ± 0.07 MPa strength in compressive conditions, acceptable cellular properties (72.3 ± 0.2 (%control) cell viability) after 7 days, good cell attachment, and calcium deposition. Overall, the results revealed that this scaffold could be a great candidate for bone tissue engineering.

## 1. Introduction

Bone disorders such as bone fracture, osteoarthritis, and osteoporosis are some of the main issues of the elderly world population. Based on previous reports, 21% of the world population will be old by 2050, demonstrating more bone disorders and bone replacements are required than the 10% of elderly people in 2000. In this regard, bone tissue engineering using innovative biomaterials and techniques has been developed in recent years [1]. Engineered scaffolds are temporary structures to provide a suitable substrate for cells to grow and, consequently, the formation of new bone. Biocompatibility, biodegradability, and mechanical and biological properties are some of the important properties of the scaffolds [2,3]. Between various materials, aluminosilicates, especially zeolite, gained much attention in recent years. Due to the periodic arrangement of channels and cages in zeolite, it has been used in different industrial applications such as catalysts, chromatography adsorbents, and ion exchangers [4,5]. Zeolite is also a biocompatible and biodegradable ceramic without any toxic body responses. Due to these great features, it has been used in different biomedical applications such as magnetic resonance imaging [6], skin hemostats [7], drug [8] and gene delivery [9], and anticancer agents [10], especially because of its porous structure, large surface area, and zero cytotoxicity, zeolite is suitable for bone tissue engineering [11]. Zeolite classification can be arranged based on their origins and also their chemical structures. More than 10 natural zeolites and 200 synthetic zeolites have been introduced. The special application of each zeolite is related to two of its important crystalline structure parameters and chemical composition. Among various synthetic zeolites, ZSM-5 is one the most used zeolite in biomedical applications. Great cytocompatibility, ability to load different kinds of drugs and bioactive molecules, and in vitro bioactivity have been confirmed in various studies [12]. In addition, results showed that zeolite could deliver oxygen to cells, stimulate osteogenic differentiation and improve bone resorption [13]. Overall, cell delivery and tissue ingrowth are affected by high porosity and high surface area to mass ratio. Especially, open pores optimize the diffusion of nutrients and waste [14]. On the other hand, both macro and micro porosities in zeolite structure can improve the cell properties of scaffolds. Microporosities are increasing the surface area to mass ratio and consequently increase the cell properties of zeolite scaffold [15].

Different strategies have been applied to develop 3D scaffolds including lyophilization [16], melt molding [17], particle leaching “phase” inversion [18], immersion [19], electrospinning [20] and 3D printing [21]. Among the aforementioned methods, the space holder method is one of the cost-effective processing methods that have good control of the morphology and pore sizes. Due to these effective features, this method has been used for bone tissue engineering for a wide range of biomaterials. For instance, Chen et al. [22] developed titanium scaffolds using the space holder strategy for bone tissue engineering. They demonstrated the formation of porous Ti scaffold with more than 95% porosity. More importantly, elastic moduli were in the range of 4–30 GPa and in the suitable range for bone tissue engineering. According to our knowledge, the space holder strategy has not been investigated for the fabrication of 3D ZSM-5 scaffolds. Recently, ZSM-5 scaffolds have been developed using 3D printing method for bone tissue engineering applications. However, weak mechanical strength and low antibacterial properties are two important limitations of ZSM-5 scaffolds for bone tissue applications.

Surface coating is a promising strategy that has been widely applied to simultaneously overcome the weak mechanical properties and improve the degradation rate and biological properties of scaffolds [23,24]. Between them, polymeric coatings are promising due to biocompatibility, biodegradability, similarity to the extracellular matrix structure, and good cell affinity [14,23,24]. Among different natural polymers, alginate (Alg) is a biocompatible, biodegradable, non-immunogenic polymer with great cellular properties [25]. Mourino et al. [26] demonstrated that Alg coating could promote the antibacterial and mechanical properties of bioactive glass scaffolds without any significant change in the interconnected pores. In similar work, Keshavarz et al. [27] synthesized Alg coated bioactive glass scaffolds and found that Alg coating increased the cell viability (45.2%), ALP activity (3.4-Infection and post-operative issues are the main issues after implantation of bone scaffolds [28,29,30,31]. The incorporation of various antibiotics, including vancomycin (VA), rifampin, gentamicin, doxycycline, nafcillin, penicillin, colistin, and minocycline, is an effective method to overcome this challenge. Between them, VA is a water-soluble antibiotic that can kill most bacteria, especially methicillin-resistant *Staphylococcus aureus* (MRSA) [32]. Osteomyelitis can be effectively treated with vancomycin, a wide glycopeptide antibiotic that is active toward Gram-positive bacteria. Gram-positive bacteria are resistant to vancomycin, but not Gram-negative ones [32,33]. Minting et al. [33] developed the VA-loaded Alg coating on the surface of magnesium alloy, which was post-treated using micro-arc oxidation. They confirmed that Alg enhanced the hemocompatibility and antibacterial properties while decreasing the degradation rate of scaffolds. According to our knowledge, VA-loaded Alg coating on ceramic scaffolds has not been widely investigated. The aim of this study is to develop bone scaffolds with desired osteogenic and antibacterial properties. A ZSM-5 scaffold is fabricated using a simple space-holder strategy and consequently, herein, a VA-loaded Alg coating is developed on it to inhibit bacterial growth and promote osteogenic differentiation. Based on our knowledge, there is no study on in vitro drug release and antibacterial properties of VA-loaded Alg coating on ZSM-5 scaffolds. So, in this study, for the first time, a ZSM-5 scaffold was developed using space holder method. Moreover, to promote mechanical and cellular properties and also to provide suitable antibacterial features, Alg coating and VA loading were utilized.

## 2. Material and Methods

### 2.1. Materials

ZSM-5 zeolite powder (≥99%) was purchased from Sigma-Aldrich. Sodium alginate (≥99%) was bought from Merck, Germany. Vancomycin hydrochloride (Applichem GmbH, Germany) and sodium chloride (≥90%), and sunflower oil (≥80%) were prepared from Sigma-Aldrich. In addition, deionized (DI) water was applied in all experiments.

### 2.2. Fabrication of ZSM-5 Scaffolds

The porous ZSM-5 scaffold was developed based on a space holder strategy using NaCl (Sigma-Aldrich) with a particle size of 300–420 µm, as the spacer, according to a previous study [34]. Firstly, the initial zeolite and NaCl powders were mixed with a weight ratio of 80/20. In order to obtain a homogeneous mixture, the powders were dispersed in sunflower oil (2 wt.%) for 1 h. Consequently, the mixture was compacted in the cylindrical mold with a diameter of 10 mm under the stress of 400 MPa using a Universal Testing Machine (Hounsfield H50KS, crosshead speed = 0.5 mm/min). Then, the sintering process was performed at 950 °C for 150 min. The heating rate was kept at 3°/min. Finally, the sintered samples were immersed in deionized water for 24 h to remove all spacer powder.

### 2.3. Preparation of Drug-Loaded Alg Coated ZSM-5 Scaffolds

**Scheme 1:**
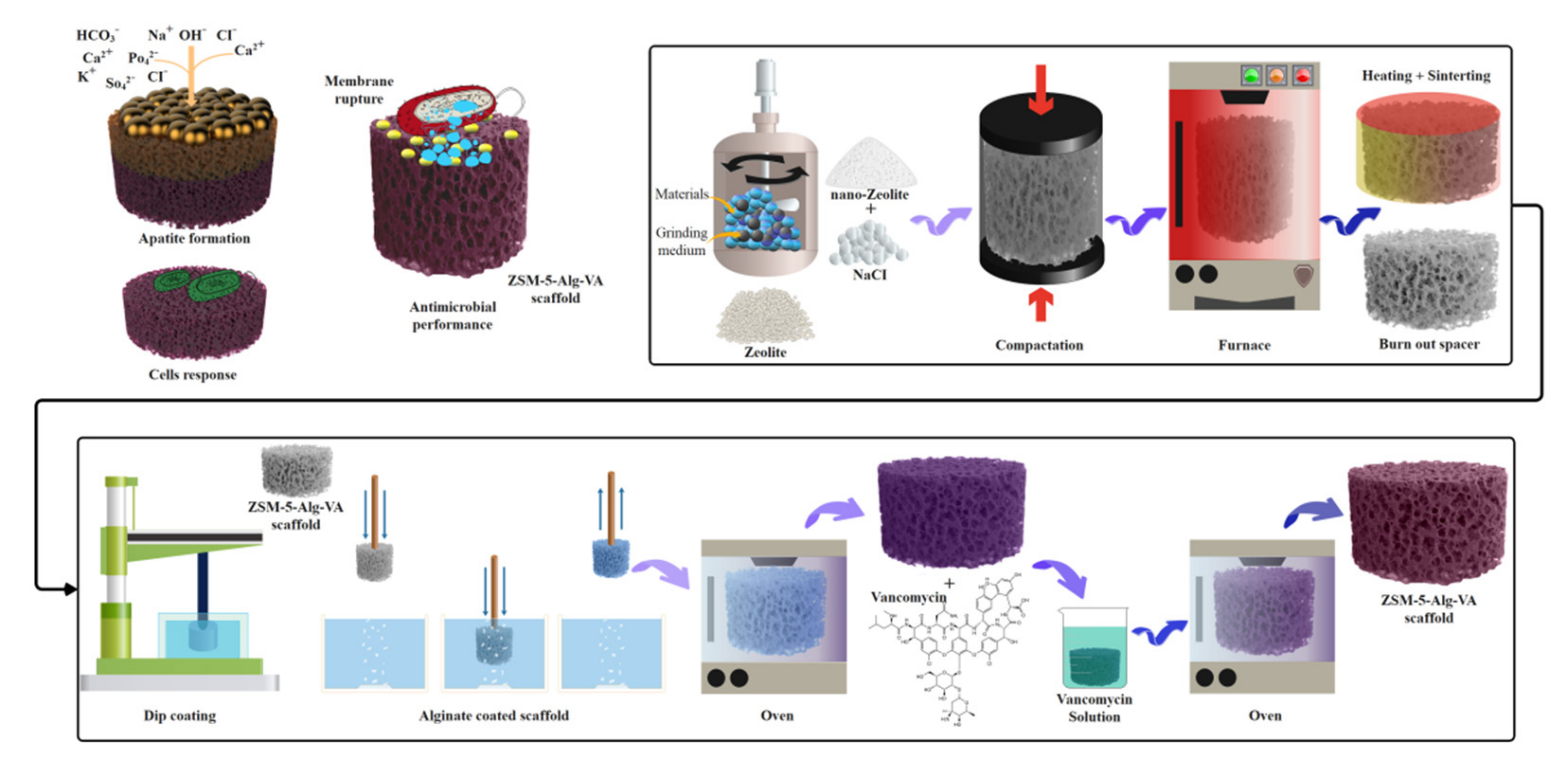
A schematic representation of the preparation and characterization of ZSM-5-Alg-VA scaffolds.

The Alg-based coating was developed using a simple dip-coating process on a zeolite scaffold. In this regard, following the preparation of 3 wt.% Alg solution, zeolite scaffolds were dipped in the Alg aqueous solution at ambient temperature for 10 min to allow penetrating Alg within the zeolite scaffold. Alg-coated scaffolds were vacuum-dried in the oven for 24 h and at 37 °C. The coating process (impregnation cycles) was repeated three times; then, the coated samples were left to dry overnight at 37 °C. In order to develop VA-loaded Alg coating, VA was dissolved in PBS with concentrations of 0.02 mg/mL, and subsequently, the scaffolds (*n* = 3) were immersed in the VA solution at 37 °C for 24 h as shown in Scheme 1.

### 2.4. Characterization of ZSM-5 Scaffolds

In order to study the surface morphology of zeolite powder, a field emission scanning microscope (FESEM, Tescan, Mira 3 Czech Republic, Prague, Czech Republic) and transmission electron microscope (TEM, Philips EM208S 100kV Netherland) were utilized. The morphology of the scaffolds was analyzed using scanning electron microscopy (SEM, Philips, XL30, Netherlands). The composition of elements was also identified using energy dispersive X-ray spectra (EDS, JSM-5910LV, JOEL Ltd., Tokyo, Japan). For the sample preparation, the scaffolds were gold-coated using a sputter coater. After imaging the cross-section of scaffolds, at least images from 3 individual samples were used to measure the pore size of scaffolds using Anix. In addition, samples were studied using X-ray diffraction (XRD, Phillips, Netherlands) and Fourier-transform infrared spectroscopy (FTIR; ALPHA-T, Bruker, Ettlingen, Germany). To evaluate the total and interconnected porosity of scaffolds, the Archimedes method was used. Furthermore, the total porosity was estimated using the Equation (1).

(1) $$\mathrm{Total}\;\mathrm{porosity}\;\%=1-\frac{W1}{\mathrm{pscaffold}\;\left(W3-W2\right)}\times 100$$

In this regard, following the estimation of the primary weight (*n* = 3, W1), the scaffolds were immersed in DI water and kept for 24 h. Consequently, the immersed scaffolds were weighed and recorded as W2. After drying, the scaffolds were weighed again, and their weight was recorded as W3 [35].

### 2.5. Machinal Properties of ZSM-5 Scaffolds

Compressive properties of scaffolds were studied with a mechanical test instrument (Hounsfield H50KS). The scaffolds (*n* = 3) were prepared in the specified dimension (8 mm diameter and 15 mm height) and consequently were fixed between two mechanical jaws and compressed with the rate of 1 mm/min based on ASTM F2150-07. From the obtained stress–strain curves, the compressive strength, and elastic modulus were determined. The elastic modulus was calculated in the strain range of 0–10% for all scaffolds and compressive strength was calculated according to the maximum stress of the stress–strain curves.

### 2.6. In Vitro Drug Release Evaluation

In order to evaluate the VA release, two samples were prepared: VA-loaded Alg coated ZSM-5 (ZSM-5-Alg-VA) and VA-loaded ZSM-5 scaffold. Three samples were fabricated from each one and soaked in PBS for 28 days. At the specific time points, 2 mL of PBS was discarded, and the absorbance of VA was measured by NanoDrop one (Thermo Fisher Scientific, USA).

### 2.7. In Vitro Bioactivity Properties of ZSM-5 Scaffolds

In order to study the in vitro bioactivity of scaffolds, they were immersed in simulated body fluid (SBF, pH = 7.4) at 37 ± 0.5 °C for 21 days. The Kokubo protocol [36] was used for the preparation of the SBF solution. After 21 days of soaking, the samples were washed with DI water and dried at room temperature. The formation of bone-like apatite on the scaffolds was determined using SEM imaging, XRD, and FTIR analysis.

### 2.8. Antibacterial Activity Evaluation of ZSM-5 Scaffolds

A disk diffusion test was utilized to determine the antibacterial properties of scaffolds using *Staphylococcus aureus* (*S. aureus,* ATCC 25923) (Ideal Gostar Co., Iran). Scaffolds were sterilized with gamma radiation, and all antibacterial assays were performed under sterile conditions and in a laminar flow cabinet. The bacterial inhibition tests were performed on the disk diffusion agar method. The *S. aureus* bacteria were added to a separate bottle of 50 mL sterile Mueller–Hinton broth and stirred. Each bottle was incubated in a shaking water bath at 37 °C for 2 h. The bacterial suspension was cooled and homogenized to room temperature. 0.5 mL of suspension was spread onto a 10 cm diameter agar. Before the sample applying, agar plates were dried at 37 °C. In order to determine the antibacterial properties of scaffolds toward *S. aureus*, samples were placed on the agar plate containing the test organism and incubated at 37 °C for 24 h to determine the inhibition zone. For further investigation, the antibacterial activity of scaffolds was studied using colony counting of bacteria in plates containing specimens. Same bacteria were used. An amount of 10 µL of bacterial suspensions was added to each test tube containing samples. These tubes were shaken at 37 °C and 160 rpm for 24 h, and in the next step, 1 mL of this suspension was diluted serially by 9 mL sterile PBS and added to a plate containing a bacterial medium. Eventually, the number of bacterial colonies formed in each plate was counted after 48 h incubation at 37 °C by a colony counter.

### 2.9. Cell Culture

MG-63 cells from the National Cell Bank of Iran at the Pasteur Institute (NCBI, C555) were used to study the cytocompatibility of scaffolds. The scaffolds were sterilized via 30 min soaking in ethanol and consequent 20 min UV exposure. The cells (10^4^ cells/mL) were seeded on the scaffolds and cultivated for 1 and 7 days in a complete culture medium based on Dulbecco’s Modified Eagle Medium (DMEM, BIO-IDEA, Iran) supplemented with 1% (*v/v*) streptomycin/penicillin (BIO-IDEA) and 10% (*v/v*) fetal bovine serum (Bioidea).

#### 2.9.1. In Vitro Cytocompatibility of Scaffolds

The metabolic activity of cells was determined using a 3-(4,5-dimethylthia-zolyl-2)- 2,5-diphenyl tetrazolium bromide (MTT) assay based on manufacture protocol (Sigma, USA). After 1 and 7 days of culture, the cell-seeded scaffolds were incubated for 4 h in MTT solution (0.5 mg/mL). Then, DMSO was applied to dissolve formed formazan crystals. The optical density (OD) of samples was determined using a microplate reader (Bio-Rad) at 490 nm. Finally, the relative cell survival (% control) was calculated using Equation (2).

(2) $$\mathrm{Relative}\;\mathrm{cell}\;\mathrm{survival}\;\left(\%\mathrm{control}\right)=\frac{X\;\mathrm{sample}-\mathrm{Xb}}{\mathrm{Xc}-\mathrm{Xb}}\;\;$$

X sample, X_b_, and X_c_ are reported absorbance of scaffolds, DMSO, and control sample, respectively [37].

#### 2.9.2. In Vitro Cell Attachment Evaluation

In order to evaluate the cell attachment and morphology, the samples were cultured for 7 and 14 days in a complete growth medium and evaluated by SEM. At the specific time point, scaffolds were fixed with 4% formaldehyde solution for 30 min. After rinsing with PBS three times, samples were dehydrated with 30, 70, 90, 96, and 100% ethanol, respectively. Finally, in the next step, scaffolds were air-dried, and gold coated. Cells’ morphology on each sample (*n* = 2) was observed by SEM (Philips, XL30, The Netherlands) [38].

#### 2.9.3. Osteogenic Differentiation Evaluation

In order to determine the calcium deposits (mineralization), Alizarin red staining was used on scaffolds after 7 and 14 days of cell culture. At the specific time points, the cells were fixed with 4% formalin for 15 min. Then, Alizarin red solution in Tris buffer (pH 4) was added to all samples. After the incubation for 1 h at room temperature, the stain was removed, and cells were rinsed with PBS. Finally, pictures were taken under the stereomicroscope (Nikon, USA). In addition, for quantitative analysis, the cells were rinsed with PBS and fixed with methanol. The samples were stained for 10 min and again washed with PBS. A total of 400 mL of 10% sulfuric acid was added to each well and sat for 30 min. After removing cellular layers, the remaining liquid was transferred to 1.5 mL microtubes. Then, for 10 min, microtubes were located at 85 °C bain-marie bath and after that immediately were located in ice for 5 min. In the next step, samples were centrifuged for 15 min in 2000 g at 4 °C. Finally, for neutralization 30 mL of 10%, ammonium hydroxide solution was added to 80 mL of extracts, and the absorbance of each sample was determined at 405 nm according to Ref. [39].

### 2.10. Statistical Analysis

Statistical analysis was accomplished via one-way ANOVA to assess statistically meaningful differences between the results according to the *p*-value < 0.05.

## 3. Results and Discussion

### 3.1. Characterization of ZSM-5 Nanopowder

**Figure 1:**
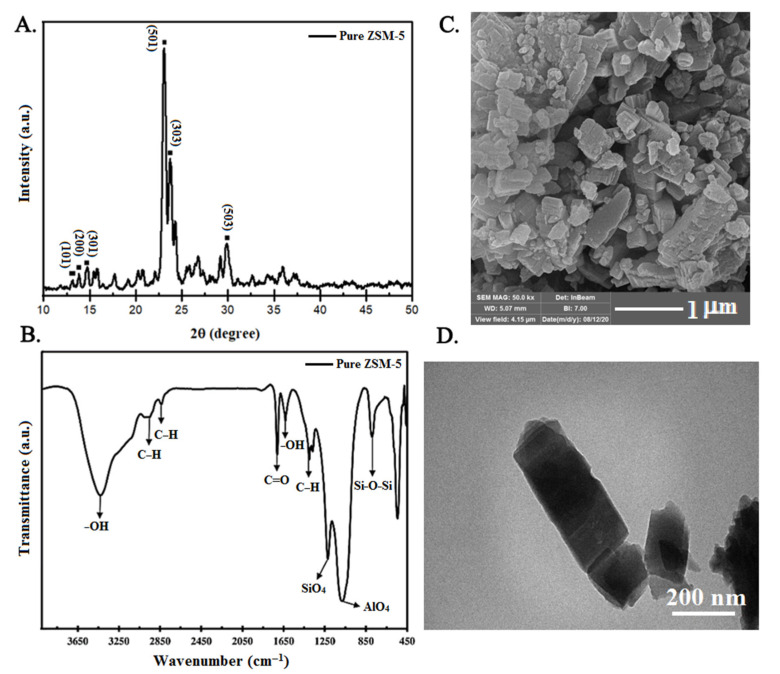
Characterization of ZSM-5 nanopowder: (**A**) XRD pattern, (**B**) FTIR spectrum, (**C**) SEM image, and (**D**) TEM image of ZSM-5 nanopowder.

In this study, the fabrication of ZSM-5 zeolite scaffolds with VA-loaded Alg coating was investigated for bone tissue engineering. The XRD pattern of ZSM-5 powder is presented in Figure 1A. Based on JCPDS 42-0024, the peaks at 2θ = 8°, 9°, 14.6°, 23°, 23.9°, and 29.6° were attributed to (101), (200), (301), (501), (303) and (503) crystallographic peaks of crystalline zeolite structure [40]. The crystallite size of ZSM-5 powder was also estimated at 22 ± 3 nm. In addition, the FTIR spectrum of zeolite powder in Figure 1B confirmed the ZSM-5 structure. The characteristic peak at 3425 cm^–1^ was related to the stretching vibration of OH bonds, while the peak at 1600 cm^–1^ was associated with the residual H_2_O in zeolite powder. Two other remarkable peaks at 1222 and 1079 cm^–1^ were related to the external vibration of SiO_4_ and AlO_4_ in zeolite structure. The stretching of the internal tetrahedra was represented at 792 cm^–1^. Finally, the fine and intense band at 540 cm^–1^ corresponded to the zeolite ring [41]. TEM and FESEM images of ZSM-5 powder are also presented in Figure 1C,D. Results showed that the ZSM-5 zeolite demonstrated two different morphologies: coffin shape crystals and boat-type crystals with a large surface area that is suitable for cellular, antibacterial, and drug delivery applications [42].

### 3.2. Physico-Chemical Characterization of ZSM-5 Scaffolds

**Figure 2:**
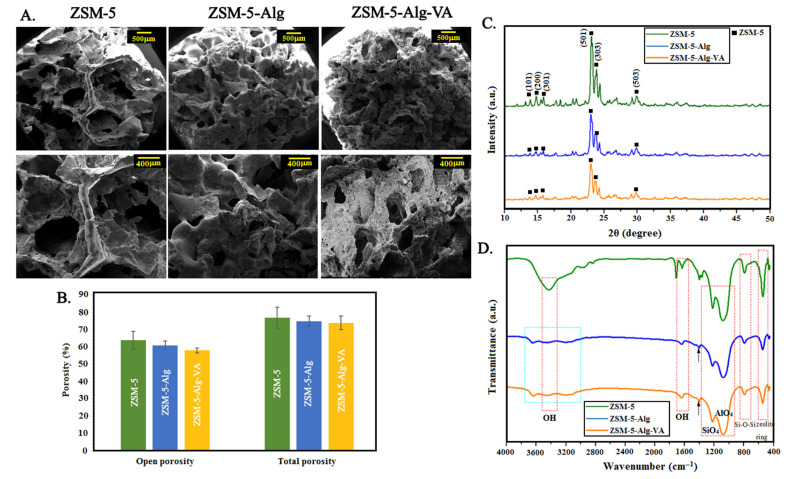
(**A**) SEM images, (**B**) open and total porosity, (**C**) XRD pattern, and (**D**) FTIR spectra of ZSM-5, ZSM-5-Alg and ZSM-5-Alg-VA.

In the next step, by adding Alg and VA-loaded Alg coating, a series of scaffolds were prepared. Engineered scaffolds should improve the attachment and proliferation of cells. Consequently, interconnected porous scaffolds are required for the transportation of growth factors and nutrients, and vascularization. SEM images (Figure 2A) demonstrated that all scaffolds possessed highly interconnected porous structures. However, the morphology of the scaffolds changed depending on the composition. The average pore size of the ZSM-5 scaffold decreased from 427 ± 2 µm to 407 ± 3 µm and 401 ± 2 µm after Alg and VA-loaded Alg coatings. Teixeira et al. [43] revealed pore sizes in the range of 200 to 500 µm could promote angiogenesis in newly formed bones. In addition, Cheng et al. [44] demonstrated that the larger pore size in the range of 250–400 µm could provide a better situation for bone formation. In another study, Lim et al. [45] showed larger pores (<3 g 500 µm) were not effective in cell attachment and osteoblast proliferation. An effective scaffold must have an interconnected porous structure in addition to porosities with a size greater than 300 µm [3]. This demonstrates that the scaffold is very permeable for tissue ingrowth, vascularization, and cell seeding. For quick protein and cell attachment, cell migration, and osseointegration, microporosity (50 < µm) is necessary. For improved new bone development, higher bone ingrowth, and capillary production, larger pore diameters (>300 µm) are necessary [23]. Abdellahi et al. [2] prepared diopside scaffolds with pore sizes ranging from 250 to 600 µm and more than 600 µm. Their result exhibited that the increase in pore size leads to the reduction of the compressive strength of the samples.

On the other hand, increasing the porosity of the structure may have negative effects on the mechanical properties and stability of scaffolds in the body. There is a critical value for pore size to balance the mechanical properties, stability, and also new bone formation and cellular properties. Zhang et al. [46] demonstrated that macro-porosities could improve the protein and oxygen delivery to new cells due to the increasing ratio of surface to volume and, consequently, increase the new bone formation. Based on the Archimedes method, the open and total porosity of structures were calculated and shown in Figure 2B. After the incorporation of Alg (ZSM-5-Alg) and vancomycin-loaded alginate (ZSM-5-Alg-VA) coatings, the total porosity did not significantly change. However, open porosity decreased in Alg and VA-Alg coated scaffolds compared to ZSM-5 scaffolds, while they are still in the suitable range for bone tissue formation. Torres et al. [28] demonstrated that after Alg coating on HA/β-TCP scaffolds, the average porosity and also the total porosity of structures decreased. A high magnification image of the Alg coated ZSM-5 scaffold demonstrated well-attachment of Alg coating on the scaffold surface and a homogenized structure. In addition, Alg coating changes the surface roughness, and more cell attachment and proliferation and, consequently, more bone formation is predictable.

XRD analysis was also used for ZSM-5 scaffolds with Alg and VA-Alg coatings (Figure 2C). All scaffolds consisted of the characteristic peaks of ZSM-5 in all samples, and no clear peaks were added after Alg and VA coating incorporation due to the amorphous structure of Alg and VA [27]. FTIR spectra of ZSM-5 scaffolds with Alg and VA-Alg coatings are presented in Figure 2D. All ZSM-5 peaks, which were described in Figure 1B, were still visible in the spectrum of scaffolds. After Alg coating, due to hydroxyl groups of Alg, two different peaks were detected at 3637 and 1639 cm^–1^. In addition, the symmetric and antisymmetric stretches of CO_2_ in Alg structure were presented at 1487 and 1403 cm^–1^ [47]. After VA incorporation, a sharp peak at 1230 cm^–1^ was related to the aromatic ester [48]. A small peak for the skeletal vibration of the CO bond at 1550 cm^–1^ and vibration of the CN group at 1062 cm^–1^ are the main characteristic peaks of VA. However, because of the integration of these peaks with Alg peaks, they were not obviously visible in the spectrum of the ZSM-5-Alg-VA scaffold. However, broadening and shifting peaks were remarkable in the FTIR spectrum, demonstrating the successful VA loading.

### 3.3. Mechanical Characterization of ZSM-5 Scaffolds

**Figure 3:**
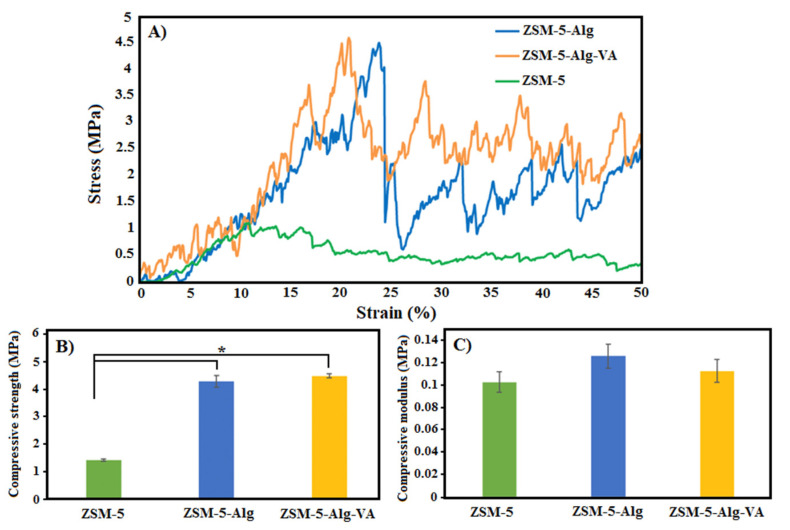
Mechanical properties of ZSM-5-based scaffolds: (**A**) compressive stress–strain curves, (**B**) the compressive strength, and (**C**) compressive modulus of ZSM-5, ZSM-5-Alg, and ZSM-5-Alg-VA scaffolds (* *p* < 0.05).

The mechanical characteristics of bone scaffolds are important properties for bone tissue engineering applications. The compressive strength and compressive modulus of each scaffold were determined according to the stress–strain curves. According to the stress–strain curves of scaffolds in Figure 3A, all samples demonstrated similar behavior during increasing strain (%), including an increase in the stress reaching the special amount of the strain (%) (10% for uncoated and 20% for coated samples) and after that decreasing. A temporary decrease in the stress was related to the thin struts cracking in the zeolite structure [49]. These results were confirmed by many other studies based on various ceramic scaffolds due to their intrinsic cracks in the ceramic structures [49,50,51]. Our results demonstrated that incorporating Alg coating into the zeolite scaffold significantly increased the compressive strength of samples from 1.44 ± 0.1 MPa to 4.3 ± 0.07 MPa, making them suitable candidates for damage bone replacement (compressive strength of spongy bone 0.2–4 MPa). The main reason for increasing compressive strength in Alg-coated scaffolds was described by Peroglio et al. [52] and Pezzotti et al. [53] as a microscale crack bridge mechanism. Based on this mechanism, polymer coating was stretched upon the crack opening and can avoid the failure of the structure. After VA incorporation in Alg coatings, the compressive strength of samples did not show significant changes (4.5 ± 0.01 MPa), and it was in the appropriate range for bone replacement. Additionally, the compressive modulus of scaffolds was not significantly changed after Alg and Alg-VA coatings, while they were in the appropriate range for bone tissue engineering. Similarly, Araujo et al. [24] demonstrated that melanin coating on bioglass scaffold increased the compressive strength from 0.5 to 1.3 MPa. The compressive modulus of bone was reported at the range of 0.1–0.4 MPa [54], which confirmed the great potential of ZSM-5 scaffolds for bone replacement. Additionally, Alg coating on the ZSM-5 matrix also had a significant effect on reducing the porosity and pore size of the scaffolds and improving the mechanical properties.

### 3.4. Drug Release Evaluation

**Figure 4:**
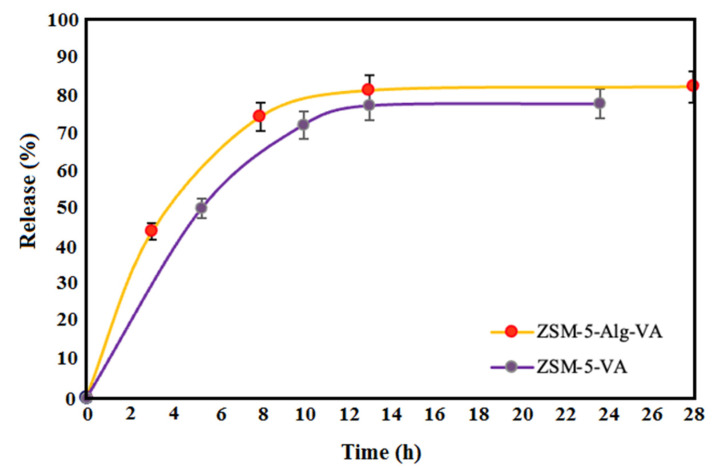
Drug release behavior of ZSM-5-Alg and ZSM-5-Alg-VA scaffolds.

In order to determine the VA release, two different samples (ZSM-5-Alg-VA and VA-loaded ZMS-5) were investigated. Figure 4 shows similar behaviors in releasing VA in two samples; in the first stage, more than 20% of VA was burst released in the first 60 min for coated scaffold and 120 min for the uncoated scaffold. It can be related to the weak physical interaction between VA and scaffolds, which happened at the loading level. In the next stage, after 10 h, the drug release in two different samples entered the plateau phase. The slow release rate of VA in the coated sample could help to prevent infection in damaged bone. In a similar study, Araujo et al. [24] showed that more ibuprofen was loaded on polymer-coated bioglass than the uncoated bioglass. Minting et al. [33] also evaluated the release of VA loading on Mg scaffolds with Alg coatings. They revealed that the first burst release happened in the first 25 min. Compared to our results, due to the zeolite structure and great interactions between zeolite, Alg, and VA, the burst release time increased to 1 and 2 h.

### 3.5. In Vitro Bioactivity of Scaffolds

**Figure 5:**
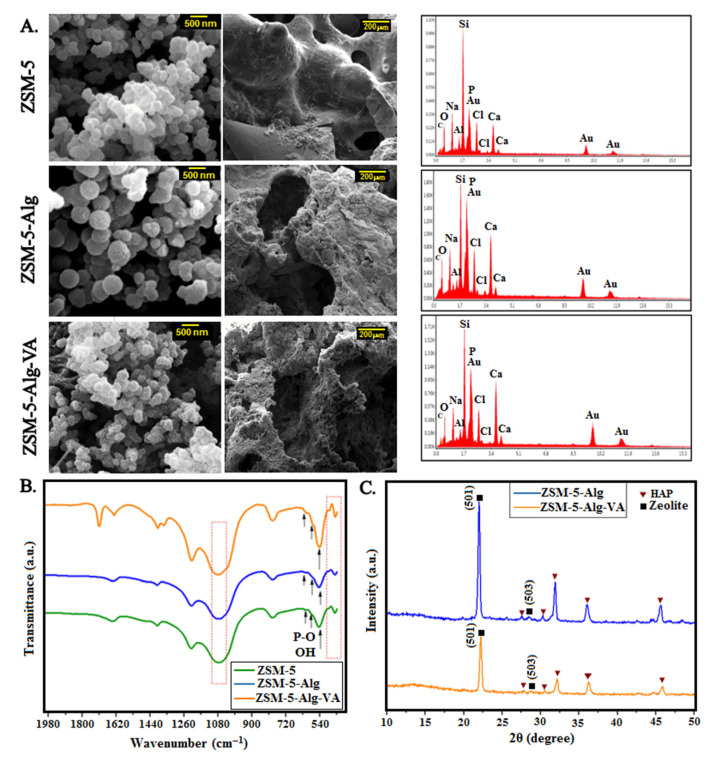
In vitro bioactivity evaluation of the ZSM-5 scaffold: (**A**) SEM images and EDX analysis, (**B**) FTIR spectra, and (**C**) XRD patterns of ZSM-5, ZSM-5-Alg, and ZSM-5-Alg-VA scaffolds after 28 days immersion in SBF.

SEM images and EDS analysis of scaffolds after 28 days of soaking in SBF are shown in Figure 5A. Deposition and the growth of bone-like apatite on the surface of samples were detected, while its density changed depending on the scaffold composition. In vitro apatite formation is a crucial step in showing new bone formation on the scaffolds. Overall, a dense and homogeneous bone-like Ca-P appeared in all samples and various coated and uncoated ZSM-5 scaffolds remained bioactive. EDS analysis also showed an increase in the Ca and P peaks and also a slight decrease in the Si peak because of bone-like apatite formation on the scaffold. The Ca/P molar ratio extracted from EDS analysis is about 1.69 in the ZSM-5 scaffold and completely confirmed the bone-like apatite formation. In similar research, Sanchez et al. [55] confirmed the bioactivity of the ZSM-5 scaffold and bone-like apatite formation on it, after 21 days of immersion. After incorporation of Alg and VA-loaded Alg coating on the ZSM-5 scaffold, SEM images and EDS analysis still confirmed the bone-like apatite formation. However, the Ca/P ratio decreased to 1.55 and 1.54, for Alg and VA-loaded Alg coating, respectively. FTIR and XRD analyses were also investigated to confirm SEM images. According to Figure 5B, FTIR spectra of both VA-loaded Alg coating and uncoated ZSM-5 after 21 days of immersion in SBF consisted of two different peaks at 580 and 617 cm^–1^ relating to the vibration of P-O bonds. In addition, the peak at 650 cm^–1^ corresponded to OH bonds confirming the bone-like apatite formation on both structures. In addition, XRD patterns of the scaffolds, after 21 days of immersion in SBF solution consisted of peaks located at 28.2°, 30.3°, 31.9°, 35.4°, and 45.5°. According to JCPDS file# 09-0432, these peaks were related to the crystalline structure of hydroxyapatite [56]. Additionally, two peaks at 28.2° and 29.6° were related to the (501) and (503) in zeolite structure, respectively as shown in Figure 5C. Due to the thickness of the bone-like apatite, some characteristic peaks of the zeolite structure did not appear in this pattern. In a similar study, Yunos et al. [57] determined the bioactivity of bioglass scaffolds with various poly-DL-lactic (PDLLA) coatings with different thicknesses. They demonstrated that, by increasing the thickness of the polymeric coating, the bioactivity of ceramic scaffolds decreased. In addition, Li et al. [58] showed the presence of vancomycin-loaded (poly(3- hydroxybutyrate-co-3-hydroxyvalerate (PHBV) coating on the bioglass scaffold can retarded the bioactivity of samples. They explained that the incorporation of polymer coatings on bioceramics and bioglass is mainly related to improving mechanical properties and drug releases, but intrinsic bioactivity has to be maintained. Based on different studies, alginate and chitosan coatings are better for maintaining the bioactivity of scaffolds [50,52,59].

### 3.6. Antibacterial Activity of Scaffolds

**Figure 6:**
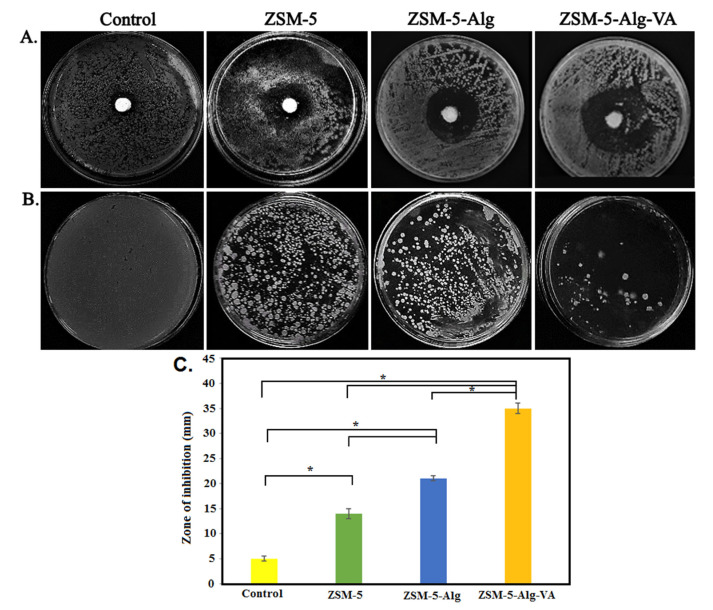
Antibacterial activity of ZSM-5 scaffolds against *S. aureus* bacteria: (**A**) inhibition zones of the ZSM-5 scaffolds with Alg and VA-loaded Alg coating, (**B**) plate count technique CFU results, and (**C**) the values of growth inhibition zones (* *p* < 0.05).

The antibacterial features of scaffolds against Gram-positive *S. aureus* were investigated. According to Figure 6, the antibacterial activity of scaffolds is enhanced after the incorporation of Alg and VA-loaded Alg coatings. A disc diffusion test (Figure 6A) demonstrated that VA-loaded Alg coating was highly able to prevent *S. aureus* bacteria proliferation. While the control sample showed an inhibition diameter of 5 mm, it was enhanced to 14 mm, 21 mm, and 35 mm, in contact with ZSM-5, ZSM-5-Alg, and ZSM-5-Alg-VA scaffolds, respectively. In similar research, Yarlagadda et al. [60] demonstrated that VA was highly active against Gram-positive bacteria, especially *S. aureus.* They pointed out that long-chain length, cationic lipophilic, and high ability to permeabilize the cytoplasmic film, are some main features of VA structure making it a great candidate for antibacterial applications. Based on the report by Minting et al. [33], *S. aureus* infections are the most reported issues after implantations. They also demonstrated that VA-loaded Alg coatings on the Mg scaffolds are a highly effective strategy to improve antibacterial activity. Figure 6B demonstrated an obvious decrease in the number of colonies on the scaffolds compared to the control group. Similar to disk diffusion results, the number of visible colonies was significantly decreased, especially after vancomycin incorporation as shown in Figure 6C. Karakecili et al. [61] demonstrated that the incorporation of VA on zeolite-coated chitosan scaffolds improved antibacterial properties and improved the scaffold responses in the bacterial situation of damaged bone.

### 3.7. In Vitro Cytocompatibility of Scaffolds

**Figure 7:**
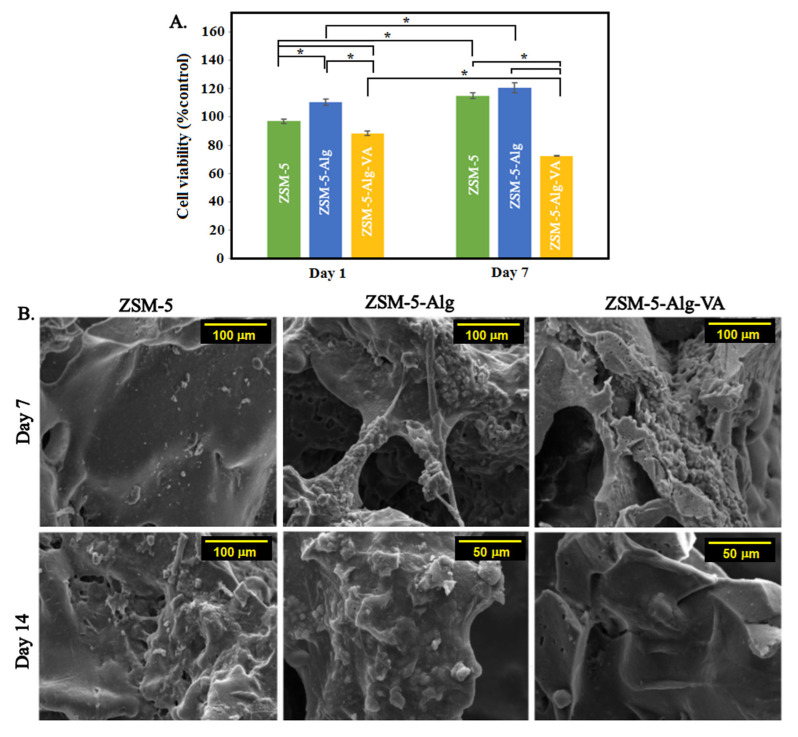
(**A**) The viability of the MG63 cells grown on ZSM-5 scaffolds with Alg and Alg-VA coatings after 1, and 7 days of culture (*n* = 3) (* *p* < 0.05), and (**B**) SEM images of the MG63 cells seeded on ZSM-5-Alg and ZSM-5-Alg-VA scaffolds for 7 and 14 days.

Osteoblast cells were used to determine the cytocompatibility of scaffolds with Alg and VA-loaded Alg coatings. In order to study the effect of coating on the cell survival rate, osteoblast cells were seeded on the coated and uncoated scaffolds, and cell proliferation was measured with MTT assay after 1 and 7 days of culture. According to Figure 7A, high cell viability after 7 days in different scaffolds approved the cytocompatibility of scaffolds. For example, after 7 days of culture, the cell viability was reported 115 ± 2.2 (%control) and 120.5 ± 3.6 (%control) for ZSM-5 and ZSM-5-Alg, respectively. In similar research, Wang et al. [62] demonstrated that Alg coating improved the cell viability on Ti implants. In research by Lee et al. [63], the main reason for increasing cell viability in the presence of Alg was pointed out. They demonstrated the similarity between the structure of Alg and extracellular matrix, and the viability of various types of cells significantly improved. After incorporating VA into the coating structure, the cell viability of osteoblast cells decreased. After 7 days of culture, the cell viability decreased from 88.4 ± 1.5 (%control) to 72.3 ± 0.2 (%control). Bakhsheshi-Rad et al. [64] reported the same results for baghdadite-VA scaffolds. They demonstrated that after 3 days of culture, the viability of MG-63 cells decreased from 77 (%control) to less than 70%. Based on their results, a high amount of VA could affect mitochondrial activity and decline the motility of cells. In another study, Rathbone et al. [65] evaluated the effect of various concentrations of different antibiotics on the activity and viability of osteogenic cells. They demonstrated a high amount of antibiotics significantly decreased the cell viability and alkaline phosphate activity compared to the control sample. Of 10 different antibiotics, VA is one of the less toxic ones and does not appreciably affect cell activity and viability. They also pointed out that drug release systems can affect the cell viability.

Cell adhesion is one of the most important parameters, especially in bone tissue engineering. Roughness, stiffness, and other surface features can affect the interaction between cells and scaffolds. According to Figure 7B, the morphology of cell-seeded scaffolds after 7 and 14 days of culture was evaluated. After 7 days of culture, osteoblast cells were attached to the zeolite scaffolds. However, Alg coating significantly improved cell adherence. Based on a similar article [27], the improved cell attachment and elongation on the alginate-coated scaffolds could be related to the similarity between the structure of coated scaffolds to the extracellular matrix, which provides a suitable and better surface for osteoblast cells. However, the cell spreading significantly reduced after the incorporation of vancomycin. It can be related to the effect of antibiotics on the cell mitochondrial activities [64].

**Figure 8:**
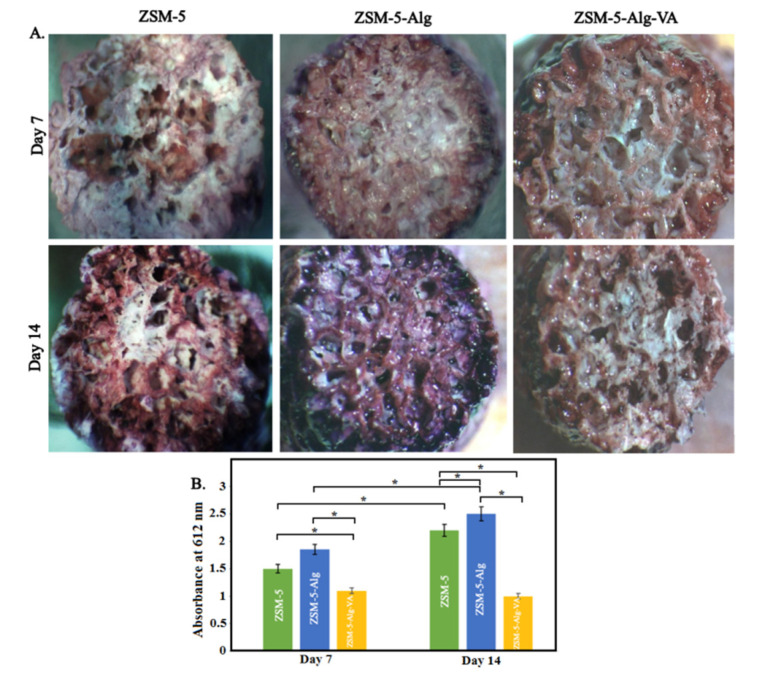
Alizarin red staining of MG-63 cells cultured on (**A**) ZSM-5, ZSM-5-Alg, and ZSM-5-Alg-VA scaffolds and (**B**) OD values of ZSM-5 scaffolds with Alg and Alg-VA coatings (* *p* < 0.05).

In order to determine the osteogenic differentiation of MG63 cells in contact with samples, Alizarin red staining was used. According to Figure 8A, red dots contributing to the calcium depositing on the cell-seeded scaffolds were spread on the surface of samples, and their intensity and density changed depending on the culture time and scaffold composition. It was clearly found that the density of these dots significantly enhanced with increasing culture time. These changes were more obvious on the Alg-coated scaffolds. Qualitative results of staining shown in Figure 8B also obviously demonstrated the great formation of calcium crystals on all samples. These results indicated the great capacity of both coated and uncoated scaffolds for new bone formation and overall, bone tissue engineering. In ZSM-5 scaffolds, due to the presence of Al and Si ions, calcium deposition was stimulated, making it appropriate for bone tissue engineering [66,67]. Wang et al. [66] demonstrated that zeolite-based coating on the 3D-printed titanium implants significantly enhanced calcium deposition. In addition, after alginate coating, calcium deposition increased in both 7 and 14 days. Similarly, various studies [68,69,70,71,72,73,74,75,76,77,78,79,80] reported that alginate coating increased calcium deposition and new bone formation. For example, Jang et al. [69] demonstrated the great calcium deposition results on the PCL scaffold with alginate coating. On the other side, after adding vancomycin to the coating structure, the calcium deposition decreased, which might be related to the negative effect of an antibiotic on cellular activities. These results were also confirmed by quantitative results from sample absorbance at 612 nm.

## 4. Conclusions

Bone damages are one of the most common health issues worldwide. Infections are also really challenging in bone problems and make bone repairs harder. Consequently, antibiotic-loaded bone scaffolds can firstly improve infection removal and, secondly, fasten bone repair. Among different materials that have been used for bone tissue engineering, bioceramics show great biocompatibility, mechanical properties, and effective cellular properties. In this study, we demonstrate the excellent effect of alginate coating on zeolite scaffolds for bone tissue engineering. Improved mechanical (4.3 ± 0.07 MPa compressive strength), cellular (120.5 ± 3.6 (%control) cell survival after 7 days), and antibacterial (inhibition zone = 21 mm) properties are some of the most useful effects of alginate coating on the zeolite structure for bone tissue applications. In addition, the incorporation of vancomycin into the alginate coating can improve the antibacterial effects of the scaffold. Despite the negative effects of vancomycin on the cellular survival rate (72.3 ± 0.2 (%control) after 7 days), they are still in the acceptable range. Taken together, vancomycin-loaded alginate coating on the ZSM-5 scaffold releases the properties that will be suitable for bone tissue engineering.

## Data Availability

All data provided in the present manuscript are available to whom it may concern.
